# Mediation of cellular osteogenic differentiation through daily stimulation time based on polypyrrole planar electrodes

**DOI:** 10.1038/s41598-017-17120-8

**Published:** 2017-12-20

**Authors:** Zongguang Liu, Lingqing Dong, Liming Wang, Xiaozhao Wang, Kui Cheng, Zhongkuan Luo, Wenjian Weng

**Affiliations:** 1 School of Materials Science and Engineering, State Key Laboratory of Silicon Materials, Zhejiang University, Hangzhou, 310027 China; 2 Zhejiang-California International NanoSystems Institute, Hangzhou, 310058 China

## Abstract

In electrical stimulation (ES), daily stimulation time means the interacting duration with cells per day, and is a vital factor for mediating cellular function. In the present study, the effect of stimulation time on osteogenic differentiation of MC3T3-E1 cells was investigated under ES on polypyrrole (Ppy) planar interdigitated electrodes (IDE). The results demonstrated that only a suitable daily stimulation time supported to obviously upregulate the expression of ALP protein and osteogenesis-related genes (ALP, Col-I, Runx2 and OCN), while a short or long daily stimulation time showed no significant outcomes. These might be attributed to the mechanism that an ES induced transient change in intracellular calcium ion concentration, which was responsible for activating calcium ion signaling pathway to enhance cellular osteogenic differentiation. A shorter daily time could lead to insufficient duration for the transient change in intracellular calcium ion concentration, and a longer daily time could give rise to cellular fatigue with no transient change. This work therefore provides new insights into the fundamental understanding of cell responses to ES and will have an impact on further designing materials to mediate cell behaviors.

## Introduction

Bioelectricity in the human body plays a key role in maintaining normal biological functions^1,2^. Reports indicated that the endogenous electric fields *in vivo* can regulate embryonic development, wound healing and neural regeneration by the transport of ionic species and macromolecules^3^. Exogenous electric field stimulation that provides an appropriate physiological environment to mimic endogenous electric fields has been utilized to manipulate transmembrane potentials to regulate cellular growth and differentiation as well as cellular functions, such as morphology, elongation, migration, and gene expression^3–5^.

A variety of cellular responses to electric stimulation of different cell types, including neurocyte, fibroblasts, osteoblasts, myoblasts, and neural crest cells have been reported^6^. For osteoblast, exposure to electrical field results in the activation of charged transmembrane receptors involving the calcium/calmodulin pathway^7^. Electrical stimulation (ES) has been shown to significantly enhance osteoblast adhesion and growth, cell proliferation^8^, mineralized nodule formation^9–11^, and the extra cellular matrix (ECM) protein synthesis^12^. It also upregulated expression of the osteoblast-specific markers (ALP, Runx2, collagen type I and OCN)^9,10,12^ and cytokines (BMP-2, IGF-1, VEGF)^13^.

To effectively mediate the cellular proliferation, gene expression and differentiation, the appropriate stimulated parameters including stimulation time, amplitude (voltage), type, and applied state of the electric field should be selected^13,14^. Because with these stimulation parameters, electrical field always affect cellular function by the change of transmembrane potential and difference in intracellular and extracellular ionic concentrations^3^, open of voltage-gated ion channel^15^, and by the generation of reactive oxygen species (ROS) in cells^16^.

Among these parameters, the stimulation time employed in the common ES is in the form of frequency, pulse duration, daily stimulation time and stimulation days. The daily stimulation time that indicates the interacted duration of ES with cells per day has been widely used as an essential parameter in the previous ES reports for mediating osteoblast function.

Ercan *et al*.^10^ used daily stimulation time of 1 h/d with 15 V constant biphasic electrical pulses on anodized titanium to improve proliferation and long-term functions (alkaline phosphatase and collagen type I synthesis and calcium deposition). Clark *et al*.^17^ employed daily stimulation time of 2 h/d with 20 mV/cm rectangular pulses of 50% duty cycle to up-regulate mRNA expression of a number of transforming growth factor (TGF)-β family genes, fibroblast growth factor (FGF)-2, osteocalcin (BGP) and alkaline phosphatase (ALP). Shao *et al*.^18^ utilized daily stimulation time of 4 h/d with the 100 μA constant current values on PLA/MWCNTs nanofibers to enhance the cellular elongation and proliferation. Zhang *et al*.^19^ used daily stimulation time of 4 h/d with the 200 μA DC stimulation on polypyrrole (Ppy)/chitosan film to increase osteoblast metabolic activity. Supronowicz *et al*.^20^ utilized daily stimulation time of 6 h/d with 10 μA alternating current stimulation on polylactic acid and carbon nanotubes to increase cell proliferation and concentration of extracellular calcium and upregulation of mRNA expression for collagen type-I. He *et al*.^21^ used daily stimulation time of 12 h/d with 10 μA constant current on Ppy nanowires to positive regulate the functions of MC3T3-E1 (cell adhesion, proliferation and differentiation) and increase alkaline phosphatase (ALP) activity, bone-carboxyglutamic acid-containing protein (BGP) and calcium deposition. Kim *et al*.^22^ utilized continuous stimulation time treatment of 1.5 μA/cm^2^ biphasic electric current on gold plates to significantly increases cell proliferation and induce the production of VEGF. Santos *et al*.^23^ used two consecutive daily cycles of 3 μA direct current stimulation on diamond-graphite nanoplatelet to enhance cell proliferation and ALP activity.

It is clear that all of these studies yielded better results for osteoblast function with daily stimulation time as compared to the control group. However, the diverseness of other ES parameters (such as voltage, ES method, electrode materials and ES types) applied in different reports make it hard to understand the mediation of daily stimulation time to osteogenic function. Meanwhile, only a few studies used daily stimulation time as an adjustable parameter to optimize the ES conditions. Wechsler *et al*.^24^ exposed adult human mesenchymal stem cell to alternating electric current with daily stimulation time of 1, 3, 6, 24 h/d (10 μA, 10 Hz frequency, sinusoidal waveform) for up to 7 consecutive days. The early gene of TAZ exhibited the highest expression after the shortest duration of 1 h/d for 1 day. Exposure of hMSCs to 3 h daily and 6 h daily stimulation resulted in a similar trend of gene expression under a sinusoidal current condition. Zhu *et al*.^25^ exposed bone mesenchymal stromal cells (BMSCs) to the daily stimulation of 0.5, 1, 1.5, 2, 2.5 and 3 h per day with a frequency of 100 Hz (duty cycle 50%) and voltage at 1 V on PLLA/3% CNT nanofibrous membrane to determine the proper duration applied in cell culture under ES. We therefore concluded that daily stimulation time is a vital parameter for mediating osteoblast function, and it is necessary to further understand the mediation of stimulation time to osteogenic differentiation for offering precise control over this differentiation.

Moreover, to achieve the effective stimulation to cell by daily stimulation time, suitable ES method, electrode materials and ES types should be carefully selected.

Planar interdigitated electrode (IDE) arrays integrate two electrodes onto the same plane, which generates EFs both parallel to and above the surface of the electrode under low voltage and allows stimulation of the cultured cells in a highly reproducible and controlled manner^26–28^. Ppy has been widely used as electrode material for ES due to its biocompatibility, good electrical conductivity, high energy storage capacity and easy and flexible synthesis in a wide range of solvents^21,29,30^. The ES type of biphasic pulse decreases the risk of the accumulation of charged proteins on the electrodes and the creation of faradic products^10,22^.

In this work, we adopted Ppy IDE with biphasic pulse and focused on the interacting duration of ES to cells to investigate the role of daily ES time in mediating osteogenic differentiation of MC3T3-E1 cells. The effect of daily ES time on growth behaviors of MC3T3-E1 cells, including the proliferation and osteogenesis-related genes (ALP, Col-I, Runx2, and OCN), was assayed, and its possible mechanism was also discussed.

## Results

### Morphology of Ppy IDE

**Figure 1:**
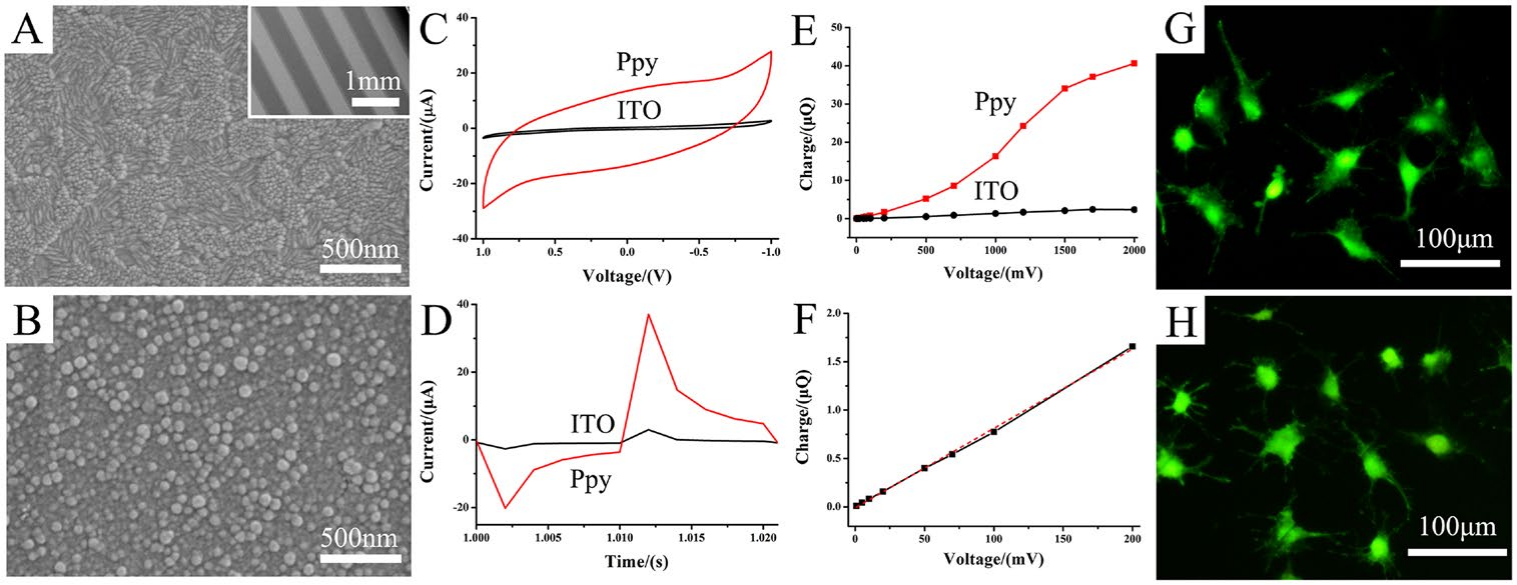
The characterization of Ppy deposited on ITO using electrochemical method. (**A**) morphology of ITO; (**B**) morphology of Ppy; The insert in A shows the morphology of IDE (both electrode width and spacing between the electrodes of IDEs was 500 μm). (**C–F**) The charge storage of Ppy IDE characterized by electrochemical measurements. (**C**) cyclic voltammetric (CV) measurement; (**D**) current-time curve collected with a pulses signal; (**E**) the charged charge-voltage curve of Ppy IDE calculated from the time integral of the current in D; (**F**) the charge-voltage curve at low voltage range. (**G** and **H**) showed the adhesion and spreading of MC3T3-E1 cell on ITO electrode and on Ppy electrode after 1 day of culture, respectively.

Figure 1A,B show the morphology of PPy IDE films that electro-formed on ITO electrode with 5 mA/cm^2^ current density for 60 s. The PPy film was formed on ITO electrode homogeneously, which consisted of nanoscale spherical-like polymeric particles with diameters of 50–80 nm.

### Charge storage of Ppy IDE

Figure 1C presents the cyclic voltammetry (CV) curves of Ppy and ITO IDE in culture medium within the voltage window of −1 to 1 V. The result showed that the CV profiles of Ppy IDE exhibited a quasi-rectangular shape CV curve indicating good capacitive behavior and high-rate capability^31^. Moreover, the curve enclosed area of Ppy IDE was larger than that of ITO electrode, demonstrating the higher charge storage (capacitance) of Ppy.

Figure 1D indicates the current-time curve collected with a biphasic pulses signal by CHI 660D. The curves showed that the charging current of Ppy was higher than ITO at the same stimulated voltage. Figure 1E,F showed the charge-voltage curve of Ppy IDE, in which charge injected onto the electrode was calculated from the time integral of the current in one period of the curve at a voltage. The charging charge on Ppy and ITO electrode increased with pulse voltage, and the charge on Ppy electrode was higher than that on ITO electrode. The results of electrochemical measurement indicated that the Ppy IDE presented high charge storage (capacitance).

According to the charge-voltage curve, three different stimulation voltages of 15, 500, 1200 mV corresponding to the selected charge values (0.1, 5 and 25 μQ) were utilized for further ES.

### Cell Proliferation under electrical stimulation

Firstly, we studied the cell viability on Ppy IDE staining by calcein-AM after 1 day of culture (Fig. 1G,H). Compared with the viable cells on ITO electrode, that on Ppy IDE also showed the excellent biocompatibility for cellular adhesion, spread and viability of MC3T3-E1 cells.

**Figure 2:**
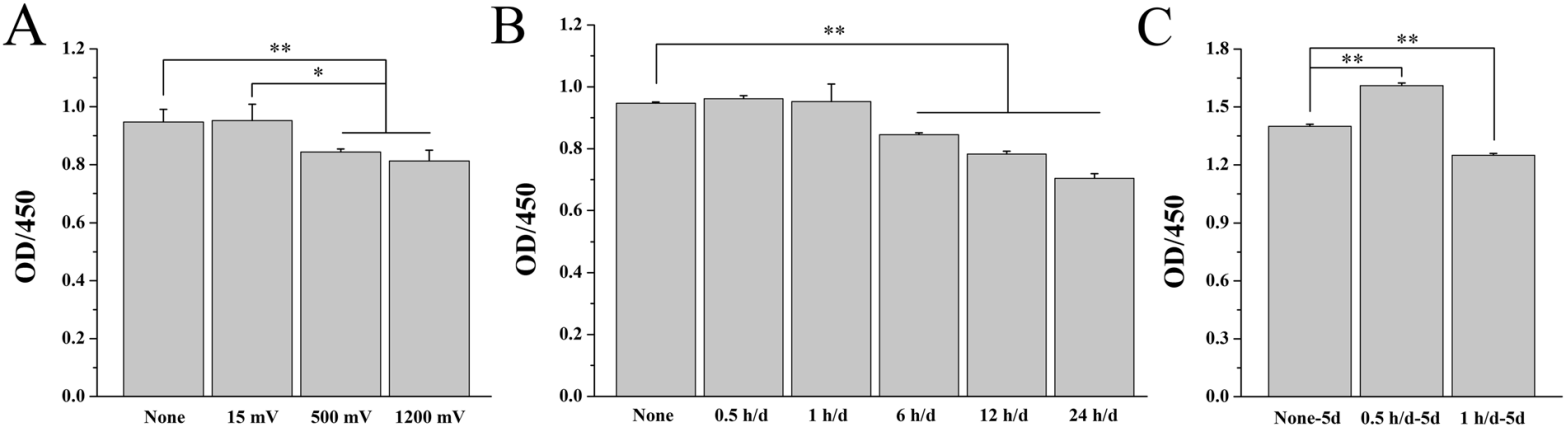
Cell proliferation on Ppy IDE under biphasic pulse stimulation with three stimulation voltages (15, 500 and 1200 mV) (**A**) and different daily stimulation time (0.5, 1, 6, 12 and 24 h/d) for 2 days (**B**), and with daily stimulation time of 0.5 and 1 h/d for 5 days (**C**). *p < 0.05, **p < 0.01.

Figure 2A shows the cell proliferation under biphasic pulse for 2 days (10 ms pulse duration, 1 Hz frequency, 1 h/d) with three different voltages (15, 500, 1200 mV) presented by the voltages according to the charge-voltage curve. The results indicated that the cell proliferation decreased with voltage increasing. Interestingly, cell proliferation under voltage of 15 mV stimulation did not decrease. Therefore, the voltage of 15 mV was selected for the further studies.

Figure 2B shows the effect of daily stimulation time (0.5, 1, 6, 12 and 24 h/d) on cell proliferation after 2 days of stimulation with 15 mV. The cell proliferation decreased significantly when prolonged the daily stimulation time. However, it is noteworthy that the daily stimulation time of 0.5 h/d and 1 h/d did not inhibit the proliferation obviously.

Figure 2C shows the effect of daily stimulation time (0.5 h/d and 1 h/d) on cell proliferation after 5 days of stimulation. Compared with the 2 days stimulation, the proliferation after 5 days of stimulation changed significantly, which presented that the proliferation increased in 0.5 h/d group whereas decreased in 1 h/d group.

Therefore, the results indicated that the proliferation of osteoblast was time-dependent, including the daily and total stimulation days.

### Cell cytoskeleton after stimulation

**Figure 3:**
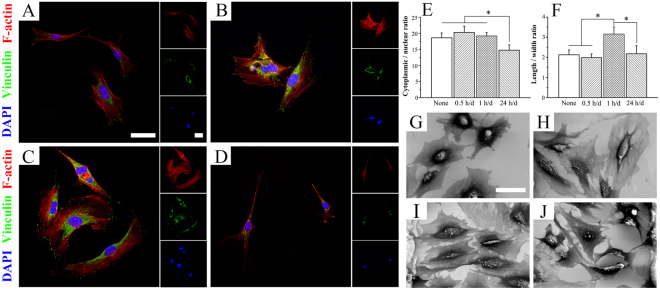
Typical FA and cytoskeleton immunofluorescence evolution of cells with daily stimulation time of 0.5 h/d (**B**), 1 h/d (**C**) and 24 h/d (**D**) after 2 days of culture. Cells were stained for the actin cytoskeleton (red), the FA protein vinculin (green), and cellular nuclei (blue). Quantitative analysis of the ratio of cytoplasmic to nuclear area (**E**) and of length to width of cytoplasm (**F**) according to the immunofluorescence staining. *p < 0.05. The morphologies of cells on Ppy IDE with no stimulation (**G**) and with the daily stimulation time of 0.5 h/d (**H**), 1 h/d (**I**) and 24 h/d (**J**) for 2 days of culture. The images share the same scale bar of 50 μm.

According to the fluorescence microscopy observation in Fig. 3, the typical morphology of MC3T3-E1 cultured on the Ppy IDE substrates with different daily stimulation time was obviously different. Cells in the daily stimulation time of 0.5 h/d group revealed more and larger focal adhesions than those of control group (Fig. 3B), as marked by vinculin, and presented a larger distribution of the actin cytoskeleton. A noticeable filopodia extensions and larger cell elongation was observed in the 1 h/d group (Fig. 3C), indicating the high potential for enhancement of osteogenic differentiation of MC3T3-E1 cells. However, after the ES with daily stimulation time of 24 h/d (Fig. 3D), cells showed smaller distribution of the actin cytoskeleton, and no obvious focal adhesion was found.

The quantitative results in Fig. 3E indicates that daily stimulation time of 0.5 h/d slightly increased the ratio of cytoplasmic to nuclear area (*p* > 0.05), whereas 24 h/d significantly decreased the ratio (*p* < 0.05). And the ratio of length to width of cytoplasm (Fig. 3F) shows the significant enhancement of elongation of MC3T3-E1 cells under daily stimulation time of 1 h/d (*p* < 0.05). Similarly, the morphologies of cell on substrates with different daily stimulation time were observed by SEM, which indicate that Ppy IDE was suitable for cell spreading (Fig. 3G). The 1 h/d group showed the obvious cell elongation (Fig. 3I), while 24 h/d group showed the smallest cell spreading (Fig. 3J).

### Osteogenic differentiation of MC3T3-E1 cells

**Figure 4:**
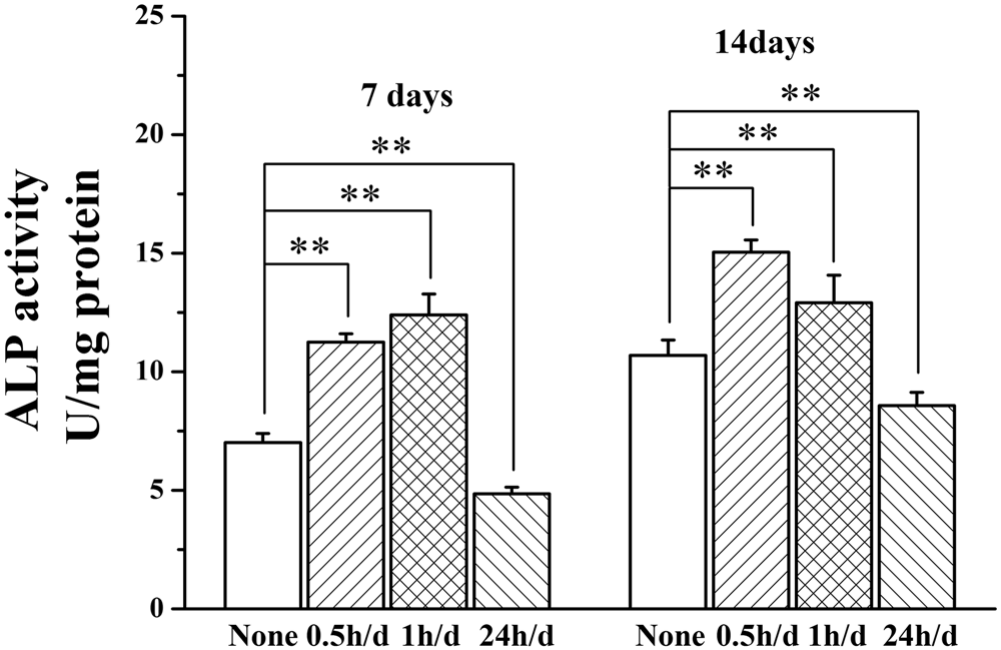
The osteoblast differentiation of MC3T3-E1 cells under the stimulation with daily stimulation time of 0.5, 1 and 24 h/d for 7 and 14 days. **p < 0.01.

Figure 4 shows the effect of daily stimulation time on osteoblast differentiation with 0.5, 1 and 24 h/d. Compared with the non-stimulated Ppy IDE, the ALP in stimulation time with 0.5 and 1 h/d increased significantly after 7 and 14 days, while that of in daily stimulation time with 24 h/d decreased.

### The expression of Ca^2+^ signaling pathway related genes

**Figure 5:**
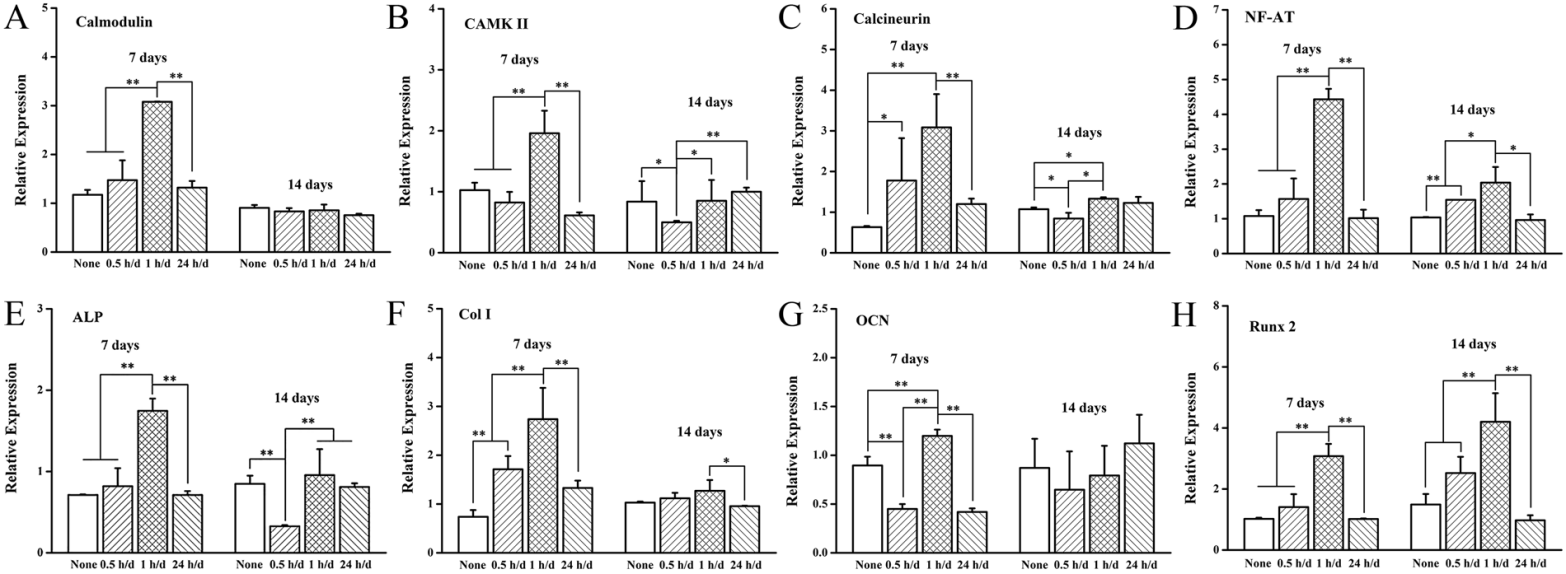
Expressions of Ca^2+^ signaling pathway related genes (CaM, CaN, CaMK II and NFAT) and osteogenesis-related genes (ALP, Col-I, Runx2 and OCN) on Ppy IDE with daily stimulation time of 0.5, 1 and 24 h/d for 7 and 14 days. *p < 0.05, **p < 0.01.

Figure 5A–D shows the gene expression of Ca^2+^ signaling pathway, which including CaM (Fig. 5A), CaMK II (Fig. 5B), CaN (Fig. 5C) and NFAT (Fig. 5D). The results revealed that the daily stimulation time of 1 h/d was observed to enhance almost every Ca^2+^ signaling pathway expression of gene. Compared with the non-stimulated group (None), the expressions of the upstream gene of CaM and CaN were upregulated by the all ES group on 7 days. However, the ES showed minor effect on gene expression on 14 days, especially to the expression of CaN and CaMK II. And the expression of upstream gene of NF-AT after stimulation of 7 days and 14 days presented the same tendency, which showed the short time stimulation (0.5 and 1 h/d) enhanced gene expression, while the long time stimulation did not influence the gene expression.

### Expressions of osteogenesis-related genes

Figure 5E–H shows the results of quantified expressions of osteogenesis-related genes, including ALP (Fig. 5E), Col-I (Fig. 5F), OCN (Fig. 5G) and Runx2 (Fig. 5H) under the various daily stimulation time by real-time PCR. The results indicated that the daily stimulation time of 1 h/d was observed to enhance almost every expression of osteogenesis-related gene to various degrees, which was consistent with the results of gene expression of Ca^2+^ signaling pathway. The daily stimulation time of 0.5 h/d and 24 h/d significantly enhanced the genes expressions of Col I at 7 days whereas that of ALP was not obvious, which were the marker of osteogenesis-related gene of early stage. However, the expression of OCN, an indicator in the later stage of osteogenic differentiation, decreased significantly under the daily stimulation time of 0.5 h/d and 24 h/d compared with the non-stimulated group. The expression of Runx 2, an essential transcription factor for osteoblast differentiation and bone formation^32^, showed the obvious daily stimulation time-dependent relationship. The daily stimulation time of 0.5 and 1 h/d enhanced the expression of Runx 2 at 7 and 14 day, whereas decreased the expression when prolonged the time to 24 h/d.

Therefore, considering the results showed above, it could be suggested that the differentiation of osteoblast can be mediated by the daily stimulation time, which might be regulated through the expression of Ca^2+^ signaling pathway and subsequently regulating the expression osteogenesis-related genes.

### Measurement of Intracellular Ca^2+^

**Figure 6:**
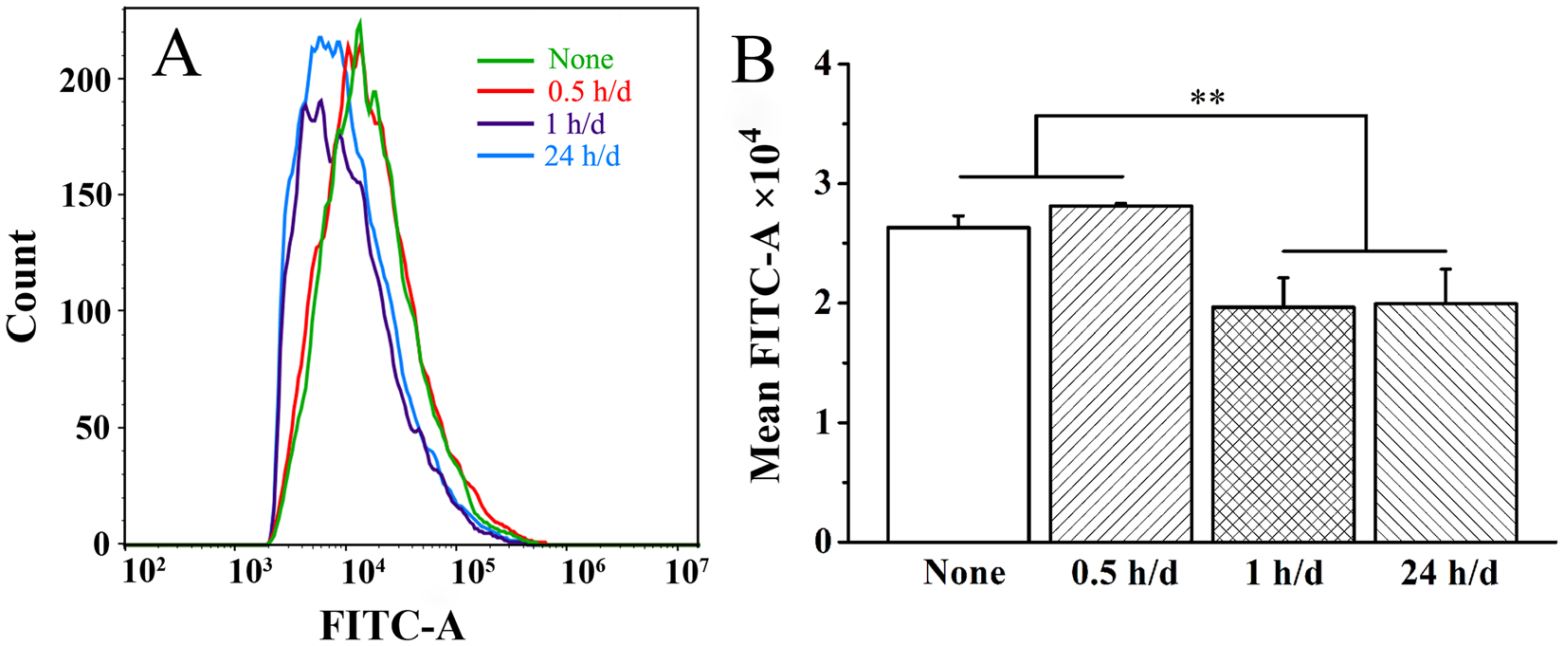
Intracellular Ca^2+^ levels recorded by flow cytometer after 2 days of electrical stimulation for 0.5, 1 and 24 h/d using Fluo4-AM calcium indicator. A: fluorescence intensity of intracellular Ca^2+^ levels; B: mean fluorescence intensity of intracellular Ca^2+^ levels. **p < 0.01.

Figure 6 shows the fluorescence intensity measured by flow cytometer after 2 days of ES for 0.5, 1 and 24 h/d. Generally, the fluorescence intensity was thought to approximately reflect the intracellular Ca^2+^ concentration^33^, which labeled by Fluo 4-AM calcium indicator that exhibited increasing large fluorescence intensity on binding cytoplasmic Ca^2+^. The flow cytometry assay showed that mean fluorescence intensity (MFI) in 0.5, 1 and 24 h/d groups was 28125.5 ± 196, 19642 ± 2466, 19924 ± 2890, respectively. Compared with the non-stimulated group (MFI 26296.5 ± 1000.6), the MFI in 1 h/d and 24 h/d group declined significantly (*p* < 0.01), whereas 0.5 h/d group presented a similar MFI. The results demonstrated that the intracellular calcium levels under interfacial ES is daily stimulation time-dependent, which indicated the higher concentration change in 1 h/d groups.

## Discussion

The Ppy IDE functioned very efficiently as an interfacial ES to cells, which could be attributed to its good deposition on ITO (Fig. 1B) and high charge storage than ITO (Fig. 1C–F). Ppy has been proved to provide suitable substrates for supporting cellular attachment, proliferation and differentiation directly through polymer-cell interaction^34–36^. And it was used as electrodes to increase the proliferation of osteoblast^21^ and smooth muscle cells when ES applied^37^. This study indicated that Ppy electrode showed the favorable cellular adhesion and viability of MC3T3-E1 cells (Fig. 1H). Cellular proliferation decreased with applied voltage from 15 mV to 1200 mV on Ppy electrode (Fig. 2A). As daily stimulation time was considered, the cell growth behavior strongly depended on the time, especially daily stimulation time. The cell proliferation decreased with daily stimulation time from 0.5 h/d to 24 h/d after 2 days of stimulation (Fig. 2B).

Reports indicated that adverse effects on cell growth were detected when the voltage was further increased and when the duration was further prolonged^25^. High voltage stimulation would overload the intercellular ions distribution and the transmembrane potential^3^, generation of relatively high field strengths and reactive oxygen species (ROS) in medium, which might be cytotoxicity to cells proliferation and even breakdown the membrane via electroporation^26^. Similarly, stimulation time means the interacting duration with cells, and a prolonged daily stimulation time might cause the long-term and continuous change in intercellular ions distribution and transmembrane potential even under the relative low applied voltage (15 mV), which was harmful to cell proliferation. Short daily stimulation time (0.5 h/d and 1 h/d) was insufficient to affect cell proliferation compared with the control after 2 days of stimulation (Fig. 2B), although the 0.5 h/d group and 1 h/d group showed to promote the filopodia extensions and cell elongation (Fig. 3). Prolonging the stimulation day to 5 days, cell proliferation presented significant response to the daily stimulation time of 0.5 h/d and 1 h/d (Fig. 2C). A better proliferation of cells might be considered as the first step of osteogenesis differentiation enhancement.

A suitable daily stimulation time of 1 h/d strongly activate calcium ion signaling pathway (Fig. 5), obviously enhance the osteogenic differentiation (Fig. 4) through upregulating expressions of osteogenesis-related genes (Fig. 5). Here, only the daily stimulation time reached a certain period, the osteogenic activity of MC3T3-E1 cells were obviously promoted, this demonstrates that the ES time is a vital factor.

Notably, according to the results of cell proliferation with variety of daily stimulation time, daily stimulation time of 24 h/d, as the largest daily stimulation and the lowest cell proliferation, was selected as an extreme stimulation condition to further understand the mechanism of stimulation time to osteogenic differentiation.

Cellular response to electrical simulation is understood as an electrical field induced change in intracellular Ca^2+^ concentrations in cytosol^38^, the changed intracellular Ca^2+^ concentration in osteoblasts will activate downstream calcineurin/NFAT signaling pathway to promote osteogenic differentiation^39^.

Since the simulation usually adopts a pulse signal with an alternative potential and cells have a strong self-regulation mechanism, the change in intracellular Ca^2+^ concentration with daily stimulation time is transient within a narrow range. While ES prolongs, the cells will fatigue to the stimulation^12^, and the intracellular Ca^2+^ concentration could have no transient change as well as no activation of the relevant signaling pathway.

For different daily stimulation time in this work, 1 h/d group and 24 h/d group showed to have similar amount of intracellular Ca^2+^ concentration, and significantly lower than that of 0.5 h/d group and control group (Fig. 6), but the early expression of calcium ion signaling pathway genes (e.g., calmodulin and NF-AT) of 1 h/d group (7 d) was significantly upregulated whereas that of 24 h/d group was almost unchanged (Fig. 5). This suggests that the upregulation of expression of calcium ion signaling pathway genes depends mainly on transient change in intracellular Ca^2+^ concentration rather than absolute intracellular Ca^2+^ concentration. It is noteworthy that the low intracellular Ca^2+^ concentration of 1 h/d group and 24 h/d group may be attributed to the special stimulation patterns of biphasic pulse. Electrical field generated by discharge and reverse charge process (negative pulse stage) was higher than initial charged process (positive pulse stage) process, which caused the calcium ions flowed out from cytosol is higher than flowed in.

**Figure 7:**
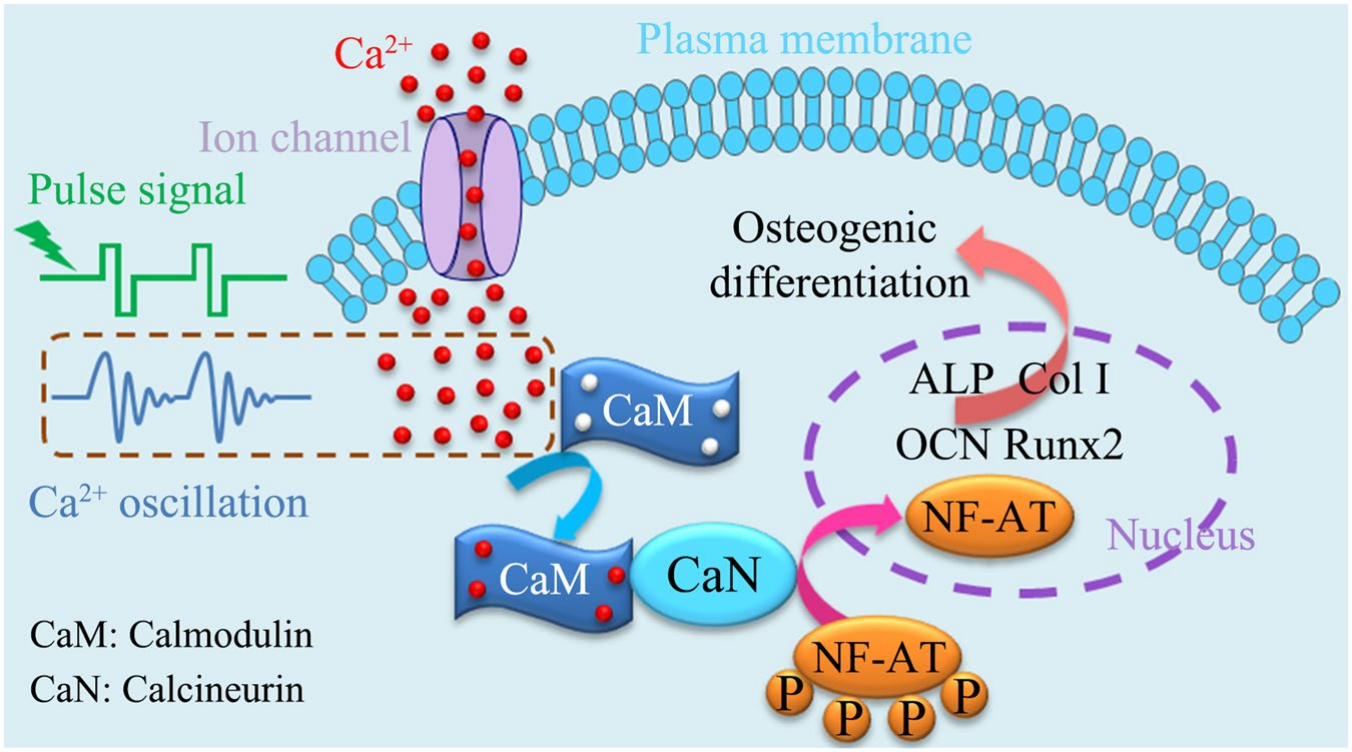
Time-dependent intracellular Ca^2+^ concentration change induced by oscillation under biphasic pulse stimulation activates the calcium ion signaling pathway by binding up four calcium ions to CaM, which upregulats the expression of osteogenesis-related gene and further mediates osteogenic differentiation.

In this work, we suggest that the time-dependent intracellular Ca^2+^ oscillation under biphasic pulse stimulation activates the calcium ion signaling pathway by binding up four calcium ions to CaM, which induces the expression of osteogenesis-related gene and further mediates osteogenic differentiation (Fig. 7). The daily stimulation time of 1 h/d is believed to receive an enough transient change in intracellular Ca^2+^ concentration, the calcium ion signaling pathway was favorably activated. Consequently, the expressions of osteogenesis-related genes were significantly upregulated and the osteogenic differentiation was obviously promoted. While 0.5 h/d group and 24 h/d group are considered to undergo insufficiently a transient change in intracellular Ca^2+^ concentration and fall in fatigue stage, respectively. Thus, the both stimulations could only result in an unobvious enhancement in osteogenic differentiation.

## Conclusions

In summary, we here demonstrated that Ppy IDE functioned very efficiently as an interfacial ES to regulate cellular adhesion, proliferation and differentiation. Increasing applied voltage and daily stimulation time decreased cellular proliferation of osteoblast after 2 days of the biphasic pulse stimulation. Daily electrical stimulation time demonstrated to obviously affect osteogenic differentiation of MC3T3-E1 cells, and a suitable daily stimulation time (1 h/d) was favorable for enhancing osteogenic differentiation of cells on Ppy IDE. The daily stimulation time could be directly related to change in intracellular calcium ions. Shorter daily stimulation time ( < 0.5 h/d) is insufficient to break the normal balance of calcium ion due to the self-adjustment of cells, while cells maintained the change induced after daily stimulation time of 1 h/d and obtained a new balanced concentration under the longer daily stimulation time (~24 h/d). Hence, properly induced the concentration change of intracellular calcium ion and activated calcium ion signaling pathway to upregulate expressions of osteogenesis-related genes.

This work reveals that a transient change in intracellular calcium ion concentration might be crucial to activate calcium ion signaling channel, and provides an insight into mediation of cellular osteogenic activity by ES.

## Methods

### Preparation of Ppy IDE electrode

ITO glass (10 mm × 10 mm) was used to prepare IDEs by laser etching. Both the electrode width and spacing between the electrodes of IDEs was 500 μm.

The electropolymerization of Ppy was conducted in a two-electrode electrochemical cell under galvanostat conditions. The polymerization solution contained 0.1 M pyrrole (Sigma-Aldrich, USA) and 0.1 M pTS (Macklin, China). Interdigital ITO was used as the working electrode and platinum sheet was used as counter and reference electrode. The polymerization current density and time was 5 mA/cm^2^ and 60 s, respectively.

### SEM observation

Field emission scanning electron microscopy (FESEM; Hitachi, S-4800, SU-70) was employed to observe the morphologies of the Ppy on ITO electrode. Specimens were washed with deionized water after electrochemical polymerization. Cellular morphology after 2 days of stimulation was also observed by SEM. The cells were fixed with 2.5% glutaraldehyde and dehydrated with gradient ethanol solutions (30, 50, 75, 90, 95 and 100 v/v % in sequence) for 10 min each^40^. All specimens were sputter-coated with a layer of gold prior to examination.

### Electrochemical testing

Cyclic voltammetric (CV) measurements were conducted by two-electrode system in culture medium with a CHI 660D electrochemical workstation (Chenhua Instrument Co., Shanghai, China) at a scan rate of 50 mV/s. One side of the Ppy IDE was served as the working electrode and another as counter electrode and reference electrode. The current-time curve was collected under a biphasic pulses signal by CHI 660D and the charge injected onto the electrode was calculated from the time integral of the current in one period of the curve. Finally, the charge-voltage curve was drawn according to the calculated data.

### Cell culture and electrical stimulation

Mouse calvaria-derived pre-osteoblastic cells (MC3T3-E1) were used in this study. Cells were cultured with alpha-modified Minimum Essential Medium (Alpha-MEM, Gibco, Waltham, MA) supplemented with 1% sodium pyruvate (Gibco), 1% antibiotic solution containing 10,000 units/mL penicillin and 10,000 μg/mL streptomycin (Gibco) and 1% MEM non-essential amino acids (Gibco) under a humidified 5% CO_2_ atmosphere at 37 °C. All cells in this study were pre-cultured for 1 day without ES to permit attachment and spreading^2,41^. Ppy IDE on glass was fixed into home-made culturing device, and then MC3T3-E1 cells were seeded at a density of 2 × 10^4^ cells/cm^2^. The ES was applied using a waveform generator (DG1022 type, Rigol Electronic Co., Ltd., Beijing, China) under a biphasic pulse with 10 ms pulse duration and 1 Hz frequency.

### Cell viability and proliferation

Cellular viability and spreading of osteoblast on Ppy-IDE after 1 day of culture was determined by a using calcein-acetoxymethylester (calcein-AM, Dojindo Laboratories, Kumamoto, Japan). Cells were washed by PBS for three times and then stained with calcein-AM of 4 μM in PBS. After 30 min of incubation at 37 °C, the live stained by calcein-AM were observed using an inverted fluorescence microscope (green fluorescence; λex = 490 nm, λem = 515 nm) (Nexcope NIB900, USA).

Three stimulation voltages of 15, 500 and 1200 mV and different daily stimulation time of 0.5, 1, 6, 12 and 24 h/d were employed for ES to investigate their effect on cell proliferation. After culture for 2 and 5 days, the viability of the cells was determined by the Cell Counting Kit-8 (CCK-8, Dojindo Laboratories, Kumamoto, Japan). Briefly, samples were transferred to a new 24-well plate and 500 μL fresh culture media and 50 μL of CCK-8 solution were added to each well and incubated for 3 h at 37 °C. The solution was dispensed into a 96-well plate, and colorimetric measurements of formazan dye were made with the microplate reader at 450 nm.

### Immunofluorescence staining

The MC3T3-E1 cells were seeded on the Ppy IDE for pre-cultured for 24 h, and then treated for 0.5 h, 1 h and 24 h per day for 2 days. The substrates were transferred to a new 24-well culture plates, and was fixed in 4% paraformaldehyde for 15 min, permeablized with 0.4% Triton × 100 in PBS, and then blocked 2% BSA/PBS solution. The fluorescent dye of rhodamine phalloidin (Phalloidin-iFluor™ 594 Conjugate, AAT Bioquest, Inc. USA) and anti-vinculin (EPR8185, Abcam, UK) was used for cytoskeleton and vinculin staining, respectively. Finally, the nucleus was stained with 4’, 6-diamidino-2-phenylindole (DAPI, ENZ-52404, Enzo Life Sciences, Switzerland). Samples were visualized by confocal laser scanning microscopy (Zeiss LSM 780, Germany). The ration of cytoplasmic to nuclear area and of length to width of cytoplasm was quantified by using software of Image-Pro Plus 6.0 system (IPP).

### Alkaline phosphatase analysis

The MC3T3-E1 cells with a density of 2 × 10^4^ cells/cm^2^ were seeded on the Ppy IDE, and treated with voltage of 15 mV for 0.5 h, 1 h and 24 h per day. After 7 and 14 days of stimulation, culture medium was removed, and the samples were transferred to a new 24-well culture plates. Then samples were rinsed with PBS for three times. The cells were lysed with CelLytic Buffer (Sigma, St. Louis), and the received cell lysate was centrifuged with the speed of 12000 rmp at 4 °C for 15 min. The supernatants were assayed by LabAssayTM ALP (Wako Pure Chemical Industries, Ltd. Japan) via measuring the optical density at a wavelength of 405 nm. The ALP activities were obtained by normalizing the quantitative assay values to total protein contents tested in a BCA protein assay.

### Quantitative Real-Time PCR assay

The expression of Ca^2+^-calcineurin/NFAT signaling (calmodulin (CaM), calcineurin (CaN), calmodulin-dependent protein kinase II (CaMK II) and NFAT) and osteogenesis-related genes (ALP, Col-I, Runx2 and OCN) was evaluated through real-time (RT) polymerase chain reaction (PCR) assay. The MC3T3-E1 cells were seeded on the samples (three replicates), and stimulated with the time of 0.5, 1 and 24 h/d for 7 days and 14 days. The total RNA was extracted using TRIzol reagent and collected using the miRNeasy Mini Kit (QIAGEN 217004, USA). RNA samples were reverse transcribed to cDNA in reactions using the PrimeScript^TM^ RT reagent Kit with gDNA Eraser (Perfect Real Time) (Takara RR047A, Japan) according to manufacturer’s protocol. The qPCR reactions were conducted on the Mastercycler® ep realplex system (Eppendorf, Germany) with a SYBR Green (PowerUp^TM^ SYBR^TM^ Green Master Mix (Applied Biosystems A25742, USA)) using 40 cycles at 95 °C for 2 min, 60 °C for for 30 s, then 72 °C for 30 s and were performed in triplicate for each cDNA. The relative expression of genes was normalized to that of the reference gene β-actin.

The primers for RT-PCR are shown in Table 1.

**Table 1:** Primers used for qRT-PCR of Ca^2+^ signaling pathway related genes (calmodulin, calcineurin, calmodulin-dependent protein kinase II (CaMK II) and NF-AT) and osteogenesis-related genes (ALP, Col-I, Runx2 and OCN). β-actin as reference gene.

No.	Gene Name	Forward primer sequence(5′-3′)	Reverse primer sequence (5′-3′)	Number of sequence on NCBI
1	Calmodulin	GGGTCAGAACCCAACAGAAG	GTCAAGAACTCTGGGAAGTCAA	NM_001313934.1
2	Calcineurin	GTAGGCACCTCACAGAGTATTT	CAGTCGAAGGCATCCATACA	NM_008913.5
3	CAMK II	GAAGAACGATGGTGTGAAGGA	AGCTGCTCTGTCACTTTGATAA	NM_177407.4
4	NF-AT	CCGTCCAAGTCAGTTTCTATGT	GTCCGTGGGTTCTGTCTTTAT	NM_198429.2
5	ALP	CCAGAAAGACACCTTGACTGTGG	TCTTGTCCGTGTCGCTCACCAT	XM_006538500.2
6	Col-I	CCTCAGGGTATTGCTGGACAAC	CAGAAGGACCTTGTTTGCCAGG	NM_007742.4
7	OCN	GCAATAAGGTAGTGAACAGACTCC	CCATAGATGCGTTTGTAGGCGG	NM_007541.3
8	Runx2	CCTGAACTCTGCACCAAGTCCT	TCATCTGGCTCAGATAGGAGGG	XM_006523545.2
9	β-actin	AATGTGGCTGAGGACTTTG	GGGACTTCCTGTAACCACTTATT	NM_007393.5

### Measurement of intracellular Ca^2+^

Intracellular Ca^2+^ levels were recorded after 2 days of ES for 0.5, 1 and 24 h/d using Fluo4-AM calcium indicator (Dojindo Laboratories, Japan). Fluo4-AM dye loading solution (3 μM in HBSS) with 300 μL was quickly but carefully added to each well. The plates were incubated at 37 °C for 30 minutes, and then in culture incubator for an added 30 min. After washing with HBSS, cells were collected by 0.5 mL trypsin. After three times of centrifugation, mean fluorescence intensity of cell suspension was measured by flow cytometer (Cytoflex, Beckman Coulter, Chin) with setting the exaction wavelength at 495 nm and emission wavelength at 518 nm.

### Statistical analysis

Three or more independent experimental specimens were used in this study for statistical analysis. All quantitative data were expressed as mean ± standard deviation (S.D.) Statistical analysis was performed using the software of Statistical Package for the Social Sciences (SPSS) version 19. In all of the statistical evaluations, *p* < 0.05 was considered as statistically significant.
